# A primary neural cell culture model to study neuron, astrocyte, and microglia interactions in neuroinflammation

**DOI:** 10.1186/s12974-020-01819-z

**Published:** 2020-05-11

**Authors:** Noah Goshi, Rhianna K. Morgan, Pamela J. Lein, Erkin Seker

**Affiliations:** 1 Department of Biomedical Engineering, University of California - Davis, Davis, CA 95616 USA; 2 Department of Molecular Biosciences, University of California - Davis, Davis, CA 95616 USA; 3 Department of Electrical and Computer Engineering, University of California - Davis, 3177 Kemper Hall, Davis, CA 95616 USA

**Keywords:** Neuron, Astrocyte, Microglia, Primary cell culture, In vitro model, Neuroinflammation

## Abstract

**Background:**

Interactions between neurons, astrocytes, and microglia critically influence neuroinflammatory responses to insult in the central nervous system. In vitro astrocyte and microglia cultures are powerful tools to study specific molecular pathways involved in neuroinflammation; however, in order to better understand the influence of cellular crosstalk on neuroinflammation, new multicellular culture models are required.

**Methods:**

Primary cortical cells taken from neonatal rats were cultured in a serum-free “tri-culture” medium formulated to support neurons, astrocytes, and microglia, or a “co-culture” medium formulated to support only neurons and astrocytes. Caspase 3/7 activity and morphological changes were used to quantify the response of the two culture types to different neuroinflammatory stimuli mimicking sterile bacterial infection (lipopolysaccharide (LPS) exposure), mechanical injury (scratch), and seizure activity (glutamate-induced excitotoxicity). The secreted cytokine profile of control and LPS-exposed co- and tri-cultures were also compared.

**Results:**

The tri-culture maintained a physiologically relevant representation of neurons, astrocytes, and microglia for 14 days in vitro, while the co-cultures maintained a similar population of neurons and astrocytes, but lacked microglia. The continuous presence of microglia did not negatively impact the overall health of the neurons in the tri-culture, which showed reduced caspase 3/7 activity and similar neurite outgrowth as the co-cultures, along with an increase in the microglia-secreted neurotrophic factor IGF-1 and a significantly reduced concentration of CX3CL1 in the conditioned media. LPS-exposed tri-cultures showed significant astrocyte hypertrophy, increase in caspase 3/7 activity, and the secretion of a number of pro-inflammatory cytokines (e.g., TNF, IL-1α, IL-1β, and IL-6), none of which were observed in LPS-exposed co-cultures. Following mechanical trauma, the tri-culture showed increased caspase 3/7 activity, as compared to the co-culture, along with increased astrocyte migration towards the source of injury. Finally, the microglia in the tri-culture played a significant neuroprotective role during glutamate-induced excitotoxicity, with significantly reduced neuron loss and astrocyte hypertrophy in the tri-culture.

**Conclusions:**

The tri-culture consisting of neurons, astrocytes, and microglia more faithfully mimics in vivo neuroinflammatory responses than standard mono- and co-cultures. This tri-culture can be a useful tool to study neuroinflammation in vitro with improved accuracy in predicting in vivo neuroinflammatory phenomena.

## Background

Neuroinflammation is present in most, if not all, pathological conditions in the central nervous system (CNS), either acting as the primary driver of these conditions or as a response to neurodegeneration or disruption of homeostasis following disease progression [1–3]. Following insult or injury to the CNS, the two primary cell types associated with neuroinflammation, microglia, and astrocytes become “activated,” as indicated by changes in their morphology and phenotype [4–6]. Previously, activated astrocytes and microglia were dichotomously classified as either neurotoxic (A1/M1) or neuroprotective (A2/M2) depending on the mode of activation; however, recent evidence suggests that both astrocytes and microglia display a wide range of phenotypes depending on the activating stimuli [7–9]. Furthermore, crosstalk between neurons, astrocytes, and microglia has been shown to play a significant role in the observed neuroinflammatory response [10, 11].

While there are a number of in vivo models to study neuroinflammation, in vitro models are often used to investigate specific molecular pathways. Current cell culture models of neuroinflammation typically consist of cultures of individual cell types with conditioned media from one cell type transferred to cultures of another cell type [12–14]. While these models have provided significant insights into neuroinflammatory processes [15], these models contain inherent limitations, most notably the inability to observe the effects of membrane-bound or cell proximity-dependent mechanisms and the fact that the concentration of secreted cytokines transferred between cultures may not be physiologically relevant. An alternative model involves seeding microglia over a previously established primary neuron culture to observe the effect of this cell-cell interaction over a short period of time (24–72 h) [16–18]. In addition to the limited time-scale of this model, the culture media used to support the microglia prior to their addition to the neuronal culture typically contains a high concentration of serum, likely causing the microglia to be in an already activated state before their addition to the neuronal cell cultures [19]. Co-cultures of neurons and astrocytes are another established method of studying neuroinflammation in vitro [20–24]. As the culture conditions for neurons and astrocytes are similar, these co-cultures can be studied over extended time scales [21, 24]. However, none of the aforementioned neuroinflammatory models are able to capture the important interplay between neurons, astrocytes, and microglia. Thus, there is a need for new, multicellular culture systems that are capable of modeling the neuroinflammatory impact of crosstalk between different cells in the CNS. This need was highlighted in a recent review on the current tools and methods for studying glia [12].

To address the shortcomings of existing in vitro models of neuroinflammation, we developed an enhanced cell culture model comprised of the three major cell types associated with neuroinflammation—neurons, astrocytes, and microglia. Primary rat cortical cells were maintained in a serum-free culture media developed to support all three cell types. We demonstrate that this “tri-culture” can be maintained for at least 14 days in vitro (DIV), without any deleterious effect of the continuous presence of microglia on the overall health of the neurons in the tri-culture. The tri-culture contains a similar relative percentage of neurons and displays a similar amount of neurite growth as compared to the microglia-free, neuron-astrocyte co-cultures. Furthermore, we demonstrate that the tri-culture system responds to several pro-inflammatory stimuli, including lipopolysaccharide (LPS), mechanical trauma, and excitotoxicity, in a manner similar to that observed in vivo.

## Methods

### Culture media preparation

Base media (plating medium and co-culture medium) were prepared as previously described [25]. Briefly, plating medium consisted of Neurobasal A culture medium supplemented with 2% B27 supplement, 1x Glutamax, 10% heat-inactivated horse serum, and 1 M HEPES at pH 7.5, while the co-culture medium consisted of Neurobasal A culture medium supplemented with 2% B27 supplement and 1x Glutamax (all from ThermoFisher). The tri-culture medium consisted of supplementing the co-culture medium with 100 ng/mL mouse IL-34 (R&D Systems), 2 ng/mL TGF-β (Peprotech), and 1.5 μg/mL ovine wool cholesterol (Avanti Polar Lipids). Due to the limited shelf life of IL-34 and TGF-β, the tri-culture medium was made fresh each week.

### General cell culture

All procedures involving animals were conducted in accordance with the National Institutes of Health Guide for the Care and Use of Laboratory Animals following protocols approved by the University of California, Davis Institutional Animal Care and Use Committee. Timed-pregnant Sprague Dawley rats were purchased from Charles River Laboratory (Hollister, CA). All animals were housed in clear plastic shoebox cages containing corn cob bedding under constant temperature (22 ± 2 °C) and a 12-h light-dark cycle. Food and water were provided ad libitum. Primary cortical cell cultures were prepared from postnatal day 0 rat pups as previously described [26]. Neocortices from all pups in the litter were pooled, dissociated, and plated at a density of 650 cells/mm^2^ on substrates precoated with 0.5 mg/mL of poly-l-lysine (Sigma) in B buffer (3.1 mg/mL boric acid and 4.75 mg/mL borax, Sigma) for 4 h at 37 °C and 5% CO_2_ then washed with sterile deionized water and covered with plating medium. Primary cortical cells were plated in plating medium and allowed to adhere for 4 h before the medium was changed to the co- or tri-culture medium. Half-media changes were performed at DIV 3, 7, and 10 with the respective media types.

### Neuroinflammatory challenges

To simulate bacterial infection, cultures were challenged with LPS (3.0 × 10^6^ EU/mg; Sigma) that was reconstituted in sterile Dulbecco’s phosphate-buffered saline solution (DPBS) with calcium and magnesium (DPBS+) (Sigma) as a stock solution of 1 mg/mL and stored at − 20 °C. Following the DIV 7 media change, each well was spiked with LPS solution to a final concentration of 5 μg/mL or an equal volume of sterile DPBS+ as the vehicle control. To simulate mechanical injury, a scratch was made in the tri- and co-cultures following the DIV 7 media change. A cross (~ 200–300-μm wide) was scratched in the center of each well using a sterile 200 μL micropipette tip. Excitotoxicity was triggered by adding varying concentrations of glutamate to the cultures. Prior to each experiment, a fresh 50 mM solution of l-glutamic acid (Sigma) in DPBS+ was prepared. This 50 mM solution of l-glutamic acid was diluted with sterile DPBS+ to 100 × stocks. At DIV 7, half of the medium was removed from each well and stored at 37 °C. The glutamate solutions were diluted 1:100 directly into the cultures; vehicle controls received an equal volume of sterile DPBS+. Glutamate-exposed cultures were incubated at 37 °C for 1 h. For co-cultures, the media collected from the cultures prior to the addition of glutamate was combined with an equal volume of tri-culture medium that had twice the concentration of supplemental factors (4 ng/mL TGF-β, 200 ng/mL IL-34, and 3 μg/mL cholesterol) to create a tri-culture medium that contained any secreted factors from the co-culture, while the stored tri-culture media was combined with an equal volume of standard tri-culture medium. Following 1 h incubation, the medium from each well was completely removed and quickly replaced with the appropriate medium type for the culture condition.

### Immunostaining

At the conclusion of an experiment, cell cultures were washed 3 times with 37 °C DPBS+ and were fixed using 4% w/v paraformaldehyde (PFA; Affymetrix) in PBS for 1 h. The fixed cells were first washed twice with 0.05% v/v Tween20 (Sigma) solution in DPBS+, followed by a 3-min permeabilization with 0.1% v/v Triton X-100 (ThermoFisher) solution in DPBS+ and two additional washes with the Tween20 solution. Samples were blocked with a solution of 5% v/v heat-inactivated goat serum (ThermoFisher) and 0.3 M glycine (Sigma) in DPBS+ (blocking buffer) for 1 h. Following this blocking step, samples were incubated for 1 h in primary antibody solution containing mouse anti-β-III tubulin (1:500 dilution, ThermoFisher), rabbit anti-GFAP (1:100 dilution, ThermoFisher), and chicken anti-Iba1 (1:500 dilution, Abcam) in blocking buffer. Samples were then washed 3 times with Tween20 solution before a 1 h incubation with secondary antibody solution containing goat anti-mouse conjugated to AlexaFluor 647 (1:500 dilution, ThermoFisher), goat anti-rabbit conjugated to AlexaFluor 488 (1:500 dilution, ThermoFisher), and goat anti-chicken conjugated to AlexaFluor 555 (1:500 dilution, ThermoFisher) in DPBS+. Following incubation with secondary antibody solution, the samples were washed 3 times with DPBS+. Lastly, samples were incubated for 5 min with a 4′,6-diamidino-2-phenylindole (DAPI) solution (1:20,000 dilution in DI H_2_O, Sigma), followed by an additional Tween20 solution wash before mounting onto glass slides using ProLong Gold Antifade Mountant (ThermoFisher). For NG2 staining, instead of mouse anti-β-III tubulin, the cultures were incubated for 1 h with mouse anti-NG2 (1:200 dilution, Abcam) along with the other primary antibodies. For f-actin staining, the slides were restained with phalloidin conjugated to AlexaFluor-555 (1:25 dilution, ThermoFisher) for 1 h following an overnight wash in PBS+ at 37 °C to remove any residual antifade mountant and an additional 5 min permeabilization with 0.1% v/v Triton X-100 solution.

### Morphological analysis

For morphological analysis, cultures were fixed with a 4% w/v PFA solution in PBS and immunostained as described above. All sample images were acquired with a Zeiss Observer D1 inverted fluorescence microscope at 100x or 200x magnification and analyzed using ImageJ. The cell number/mm^2^ of the different cell types was determined by manually counting the number of nuclei that were co-localized with β-III tubulin (neurons), GFAP (astrocytes), or Iba1 (microglia) from the 100x magnification images. The average astrocyte/microglia areas were determined by manually tracing the outline of astrocytes/microglia from 200x magnification images and determining the area inside the trace. For both of these manual quantification methods, the images were de-identified, and the investigator was blinded to the experimental group. Percent area coverage of neurons or astrocytes was determined through the use of the Huang auto-thresholding method [27] on the β-III tubulin (neurons) or GFAP (astrocytes) channel.

### Apoptosis assay

Apoptosis was quantified using the Caspase-Glo® 3/7 Assay System (Promega) according to the manufacturer’s protocol. Luminescence was measured using a H1 hybrid microplate reader (BioTek Instruments).

### Calcium imaging

Prior to imaging, tri- and co-cultures were loaded with cell-permeant Fluo-4 AM calcium indicator (ThermoFisher) following the manufacturer’s protocol. To determine the effect of glutamate on calcium fluxes in the tri- and co-cultures, each culture type was spiked with varying concentrations of l-glutamic acid (Sigma) in DPBS+ or an equal volume of DPBS+. For each well, prior to the addition of the glutamate solution, a 200x magnification fluorescence image was taken, after which the shutter to the light source was closed, and the glutamate solution was added to the well. Following a 2-min incubation in the glutamate solution, a second fluorescence image was taken over the same field-of-view with the same exposure time and was used to compare the change in fluorescence intensity following the addition of glutamate.

### Cytokine profile

Following the DIV 7 media change, co- or tri-cultures were incubated with 5 μg/mL LPS or vehicle control for 48 h. Following incubation, the conditioned media was spun down to remove any cells, and the supernatant containing the conditioned media was stored at − 80 °C until analyzed. The proteome profile was determined using a Proteome Profiler Rat XL Cytokine Array (Bio-Techne) in conjugation with the IRDye® 800CW (Bio-Techne) for use with the LI-COR Odyssey® Imaging System. Relative concentrations of each cytokine in conditioned media were determined using ImageJ to compare the total pixel intensity from each spot. Hierarchical cluster analysis was performed using the MATLAB (2019a) bioinformatics toolbox.

### Statistical methods

For all experiments, a minimum of three biological replicates was used with a minimum of three technical replicates per each biological replicate. Furthermore, unless otherwise noted, for experiments requiring image analysis, at least three predetermined fields were analyzed per technical replicate to account for variability within the culture itself. When comparing the response of the tri- and co-cultures to different treatments, a two-way ANOVA was used. If the interaction was determined not significant (*p* < 0.05), then the analysis of the main effects was used to compare the two treatments. If a significant interaction was found, analysis of the simple main effects was conducted via a post hoc Tukey test. A one-way ANOVA test was used when comparing multiple groups against a single treatment, while a two-tailed Student’s *t*-test assuming unequal variances was used when only two groups were analyzed. For all experiments, statistical significance was determined by *p*-values < 0.05.

## Results

### The tri-culture supports neurons, astrocytes, and microglia in vitro

**Fig. 1:**
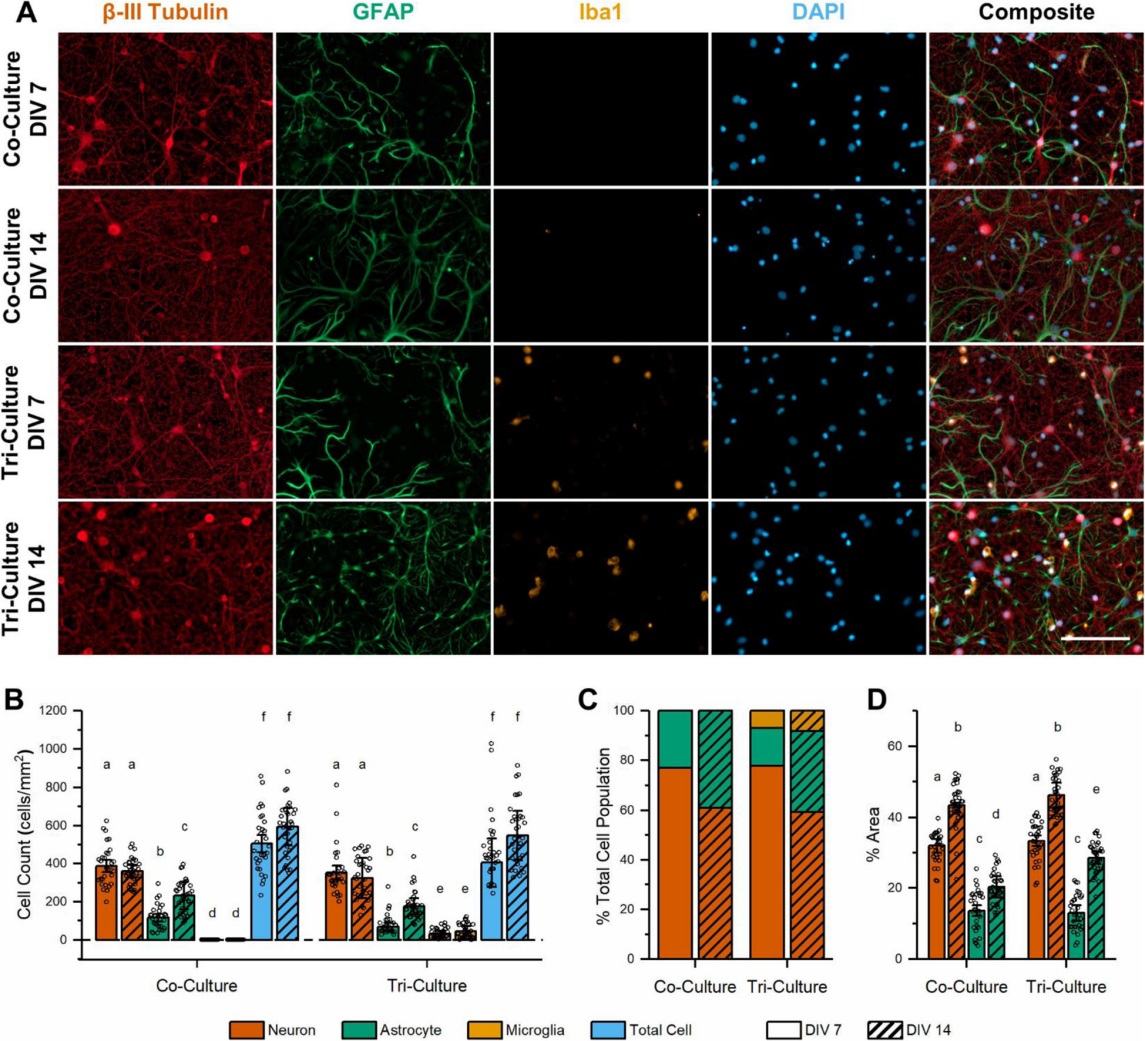
Tri-culture media is capable of supporting neurons, astrocytes, and microglia. **a** Representative fluorescence images of the tri- and co-cultures at DIV 7 and 14. The cultures were immunostained for the three cell types of interest: neurons—anti-βIII-tubulin (red), astrocytes—anti-GFAP (green), microglia—anti-Iba1 (orange), general nuclear stain DAPI (blue). Microglia are present in the tri-culture at both time points but are absent in the co-culture. Scale bar = 100 μm. **b** Quantification of the number of each cell type and total number of cells per mm^2^ for each condition shown in Fig. 1**a** (mean ± SD, *n* = 3). **c** Percent of total cell population represented by each cell type in co-culture vs. tri-culture conditions. **d** Quantification of the percent area coverage of the neurons and astrocytes in the co- and tri-cultures at DIV 7 and 14 (mean ± SD, *n* = 3). **b**, **d** The letters above the bars indicate statistically distinct groups (*p* < 0.05), while the points indicate the values of the technical replicates

Primary cortical cells taken from neonatal rats were cultured in our previously described neuron-astrocyte co-culture media [25], or in our tri-culture media consisting of the co-culture media supplemented with 100 ng/mL IL-34, 2 ng/mL TGF-β and 1.5 μg/mL cholesterol. Immunostaining for Iba1 revealed that there was a significant population of microglia at both DIV 7 and 14 in the cultures maintained in the tri-culture media, which are absent from cultures maintained in the co-culture medium (Fig. 1a–c). A two-way ANOVA did not establish a significant interaction between the time in culture and media type on the number of microglia present in the culture (*p* = 0.44). Analysis of the main effects indicated that the tri-cultures had significantly more microglia present than the co-cultures (*p* = 0.0025); however, the time in culture did not impact the number of microglia (*p* = 0.44). The total number of microglia in the tri-culture accounted for ~ 7–8% of the total cell population (Fig. 1c) in agreement with microglia numbers reported in vivo [28, 29].

Immunostaining for β-III tubulin to label neurons and GFAP to label astrocytes revealed a healthy population of both neurons and astrocytes in both the tri- and co-cultures (Fig. 1a). The number of neurons present was not affected by either the media type or time in culture (Fig. 1b, *p* = 0.44 and *p* = 0.31, respectively). Additionally, neurite outgrowth (measured as the percent of the area stained for β-III tubulin as compared to the total image area) was not statistically different between the co- and tri-cultures (Fig. 1d, *p* = 0.13), and the neurons continue to produce new projections through DIV 14 as determined by the significant increase in neuron percent area coverage from DIV 7 to 14 (*p* = 1.04 × 10^−5^). Furthermore, by DIV 14, we observed the co-localization of f-actin and β-III tubulin indicating the presence of dendritic spines (Supplementary Figure 1). As no mitotic inhibitors were added to the culture media, the number of astrocytes significantly increased in both culture types from DIV 7 to 14 (Fig. 1b, *p* = 0.0023). While the analysis of the main effects from the two-way ANOVA did not reveal a significant difference in astrocyte population between the two culture types (*p* = 0.062), the results suggest that the tri-cultures may contain a lower number of astrocytes than the co-cultures. Unlike the neurons, the astrocyte percent area coverage showed a significant interaction between the culture type and time in culture (Fig. 1d, *p* = 0.0077), with the tri-culture having a similar astrocyte percent area coverage to the co-culture at DIV 7 (*p* = 0.90), but a significantly higher astrocyte percent area coverage at DIV 14 (*p* = 0.0076).

**Fig. 2:**
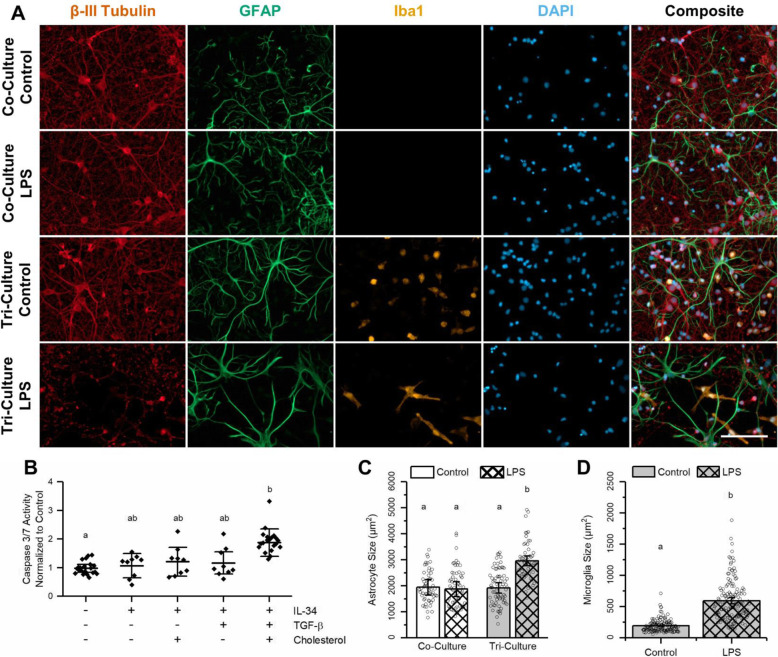
Comparing the effect of LPS on the co- and tri-culture models. Comparison of the effect of a 48-h exposure to 5 μM LPS on the co- and tri-cultures. **a** Representative fluorescence images of the tri- and co-cultures after a 48-h incubation with 5 μM LPS or vehicle. The cultures were immunostained for the three cell types of interest: neurons—anti-βIII-tubulin (red), astrocytes—anti-GFAP (green), microglia—anti-Iba1 (orange), and the general nuclear stain DAPI (blue). Scale bar = 100 μM. **b** Comparing the effects on apoptosis following a 48-h incubation with 5 μM LPS on DIV 7 cortical cultures maintained in different media types (mean ± SD, *n* = 3–6). **c** A comparison of the average astrocyte area from different conditions (mean ± SD, *n* = 3). **d** Comparing the average microglia size in tri-cultures exposed to LPS vs. vehicle (mean ± SD, *n* = 3). **b**–**d** The letters above the bars indicate statistically distinct groups (*p* < 0.05), while the points indicate the values of the technical replicates

As the three media supplements present in the tri-culture have been shown to support isolated microglia and are found in astrocyte-conditioned media [19], we were interested to see if the neurons or astrocytes might be constitutively secreting any of these factors in the tri-culture, thereby making their addition to the tri-culture media redundant. An exploratory study unambiguously indicated that IL-34 was required for microglia survival (Supplementary Figure 2); however, the additional presence of TGF-β or cholesterol did not appear to impact the number of microglia present in the culture. Additionally, neither TGF-β nor cholesterol allowed for microglia survival on their own. In order to determine if either TGF-β or cholesterol was required to maintain physiologically active microglia, we challenged cultures maintained under different combinations of TGF-β and cholesterol plus IL-34 with 5 μM of LPS. LPS is a potent activator of neuroinflammation and neuronal apoptosis, which acts through the toll-like receptor 4 (TLR4) found only on microglia in the CNS [30, 31]. As expected, LPS did not increase caspase 3/7 activity in neuron-astrocyte co-cultures relative to the vehicle control. However, cultures grown in the presence of different combinations of the tri-culture factors responded to LPS with increased caspase 3/7 activity (Fig. 2b, *p* = 0.011). In particular, cultures exposed to all 3 co-factors (IL-34, cholesterol, and TGF-β) showed a significant increase in caspase 3/7 activity following the addition of LPS (*p* = 0.0081). Cultures maintained in the co-culture medium spiked with a subset of the co-factors (IL-34 alone, IL-34 plus cholesterol, or IL-34 plus TGF-β) all showed increased caspase 3/7 activity, but a post hoc Tukey test did not reveal any significant differences between these cultures and either the co- or tri-culture. However, the fact that the change in caspase 3/7 activity of these cultures more closely resembled that of the co-culture (*p* = 0.99, 0.91, and 0.95, respectively) than that of the tri-culture (*p* = 0.064, 0.16, and 0.16, respectively) indicates that the microglia present in these cultures are most likely not physiologically active. Thus, all three factors are required to support a healthy tri-culture of neurons, astrocytes, and microglia.

### Tri-culture response to LPS

The use of LPS to stimulate a neuroinflammatory response is used by researchers to study a wide range of neuroinflammatory and neurodegenerative conditions [32], including Alzheimer’s disease (AD) [33], Parkinson’s disease (PD) [34], and even mood disorders such as clinical depression [35]. Therefore, we next determined the response of the tri-culture model to LPS. As stated previously, a 48-h incubation with 5 μM LPS significantly increased caspase 3/7 activity in the tri-culture model relative to the neuron-astrocyte co-culture (Fig. 2b). This is in agreement with previous studies that show LPS induces a neurotoxic pro-inflammatory condition both in vitro and in vivo via apoptotic caspase-3- mediated mechanisms [15, 36, 37]. Immunohistochemical analyses (Fig. 2a) reveal clear morphological changes in both astrocytes and microglia in the tri-culture after exposure to LPS. Astrocytes in the tri-culture showed reduced ramification, increased process length, and hypertrophy, all of which are hallmarks of reactive astrocyte morphology [38]. The increased process length and hypertrophy result in an overall increase in astrocyte area (Fig. 2c) that was significantly higher in tri-cultures exposed to LPS relative to tri-cultures exposed to vehicle (*p* = 0.0030) or astrocytes in the co-culture exposed to LPS (*p* = 0.0039). Additionally, there was no change in the morphology or average area of astrocytes in the co-cultures exposed to LPS or vehicle (*p* = 0.90). The size of microglia also significantly increased in tri-cultures exposed to LPS as compared to vehicle control tri-cultures (Fig. 2d, *p* = 0.0052).

### Tri-culture response to mechanical injury

**Fig. 3:**
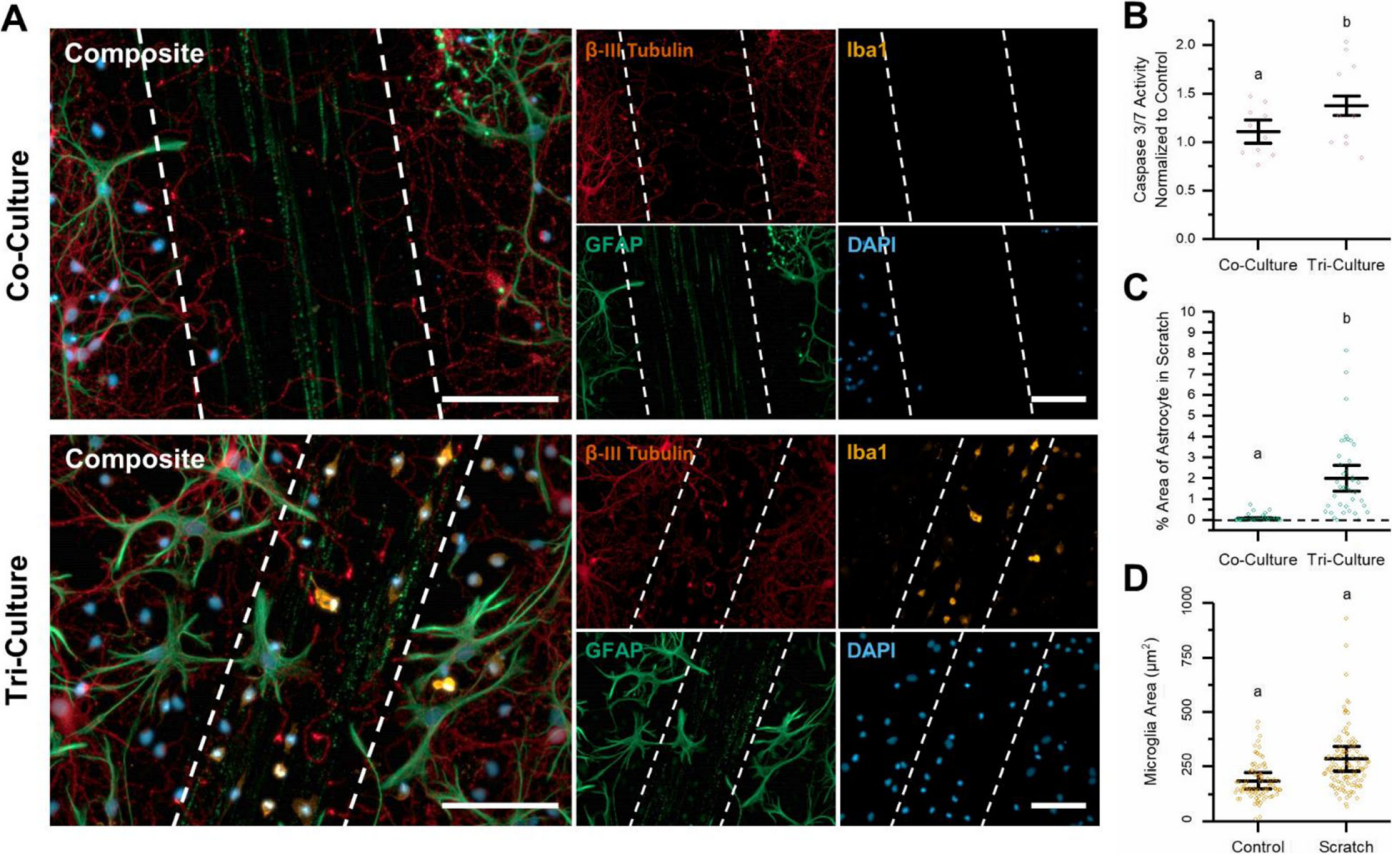
Response to mechanical injury. Comparing the effects from a simulated mechanical trauma (scratch) on co- and tri-cultures. **a** Representative fluorescence images of the co- and tri-cultures at 48 h following the simulated mechanical trauma. The dashed lines in the image highlight the area that was damaged by the scratch. The cultures were immunostained for the three cell types of interest: neurons—anti-βIII-tubulin (red), astrocytes—anti-GFAP (green), microglia—anti-Iba1 (orange), and the general nuclear stain DAPI (blue). Scale bar = 100 μM. **b** There was a significant increase in caspase 3/7 activity in the tri-culture as compared to the co-culture 48 h after the simulated mechanical trauma. **c** Comparing the percent area coverage of astrocytes in the scratched area between the co- and tri-cultures 48 h following the scratch. **d** Difference in the microglia area in the control and scratched tri-cultures. **b**–**d** All graphs display mean ± SD (*n* = 3), while the points indicate the values of the technical replicates. The letters above the bars indicate statistically significant differences (*p* < 0.05) as found by a *t* test assuming unequal variances

In order to simulate a mechanical injury, we performed a scratch assay by drawing a pipette tip through co- and tri-cultures at DIV 7. Scratch assays are a common method used to model and measure cell migration [39] and have also been used to simulate mechanical injuries, such as the trauma induced by the insertion of therapeutic implants, on cultured neurons and glial cells [40–42]. At 48 h following the scratch injury, we see a significant population of microglia migrated into the damaged area (Fig. 3a). In the tri-culture injury model, while the microglia that migrate into the injury site do not have a statistically significant larger surface area than the microglia in the control condition (Fig. 3d, *p* = 0.078), they do show a trend of having a slightly larger surface area than microglia in the non-injured tri-culture.

Glial scarring is another aspect of the inflammatory response to mechanical injury, and it consists of the formation of a glial sheath consisting mostly of reactive astrocytes that encapsulate the damaged tissue, isolating it from healthy tissue [6]. While the formation of a glial scar may take weeks to fully mature, reactive astrocytes begin to migrate towards the injury site in less than 24 h [6]. We observed this in the tri-culture as astrocytes began to migrate into the scratched area; in contrast, in the co-culture, the percent area coverage by astrocytes in the scratched area was almost non-existent (Fig. 3c, *p* = 0.032). Additionally, at 48 h following the scratch injury, caspase 3/7 activity was increased in both the tri- and co-cultures; however, the presence of microglia appeared to amplify a neurotoxic inflammatory response to the scratch, leading to a greater increase in caspase 3/7 activity in the tri-culture (Fig. 3b, *p* = .035).

### Tri-culture response to excitotoxicity

At DIV 7, tri- and co-cultures were exposed to varying concentrations of glutamate for 1 h to simulate an excitotoxic event. Following glutamate exposure, both tri- and co-cultures were maintained for 48 h in tri-culture medium to eliminate the additional factors in the tri-culture medium as potentially confounding factors in the response. In particular, TGF-β [43] and IL-34 [44] have been shown to be neuroprotective during excitotoxicity. We observed no microglia in the co-culture media at the end of the experiment, further confirming that the co-culture medium is incapable of supporting even an insignificant microglia population.

**Fig. 4:**
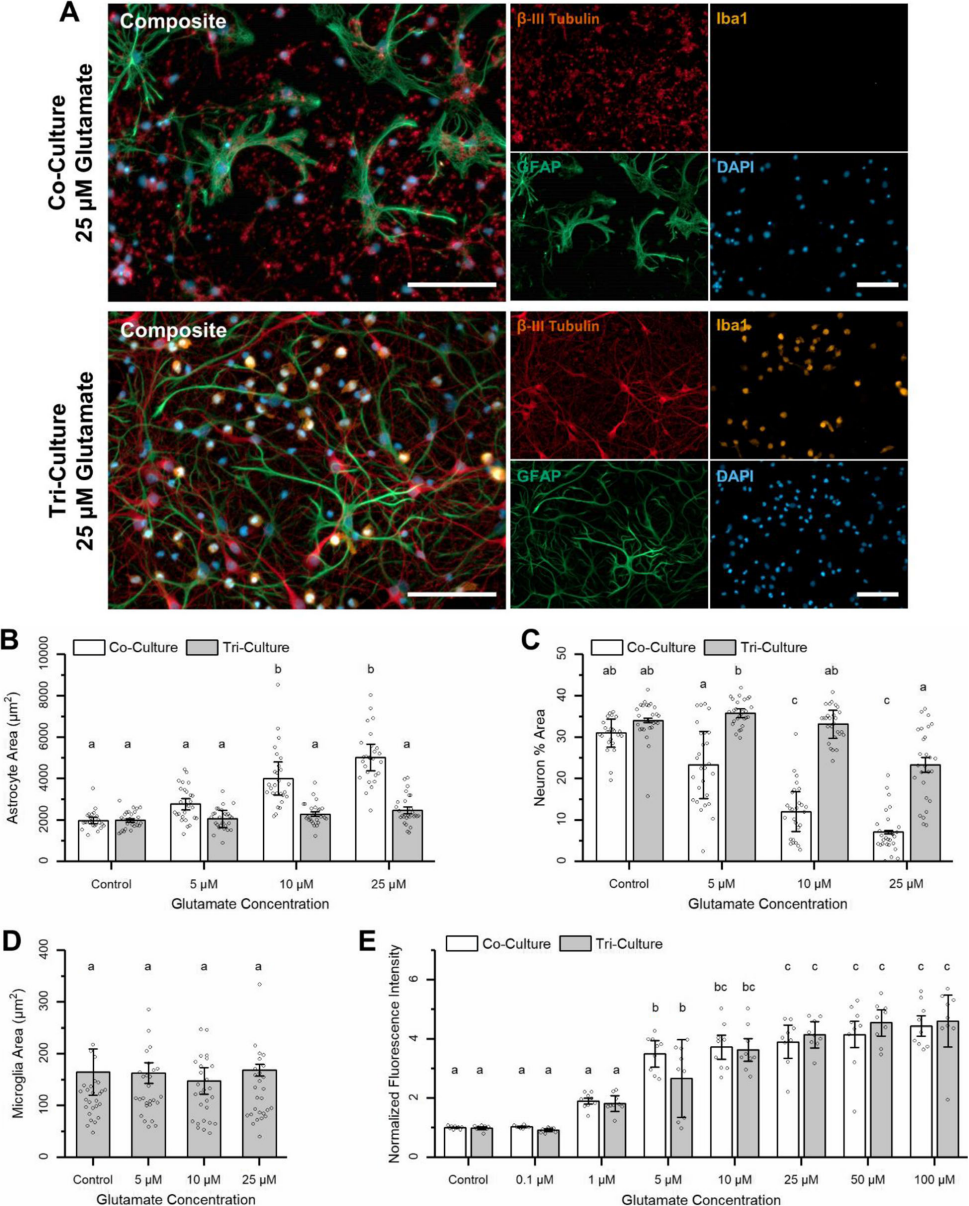
Response to excitotoxicity. **a** Representative fluorescence images of the co- and tri-cultures at 48 h following exposure to 25 μM glutamate for 1 h. The cultures were immunostained for the three cell types of interest: neurons—anti-βIII-tubulin (red), astrocytes—anti-GFAP (green), microglia—anti-Iba1 (orange), and the general nuclear stain DAPI (blue). Scale bar = 100 μM. Representative images from the other conditions can be found in Supplementary Figure 4. **b** Comparing the average astrocyte area between the co- and tri-cultures following challenges with different concentrations of glutamate. A full analysis of the simple main effects can be found in supplementary data table 1. **c** The neuron percent area coverage, with a 0.2 circularity cutoff to eliminate cell debris, following excitotoxic challenge. A full analysis of the simple main effects can be found in supplementary data table 2. **d** The average microglia surface area did not change following treatment with different concentrations of glutamate. **e** Calcium imaging results showing the change in fluorescence intensity following treatment with different concentrations of glutamate. A full analysis of the simple main effects can be found in supplementary data table 3. **b**–**e** All graphs display mean ± SD (*n* = 3), while the points indicate the values of the technical replicates. The letters above the bars indicate statistically significant differences (*p* < 0.05)

Our results suggest that microglia in the tri-culture play a significant neuroprotective role during excitotoxic events. We observed significant neuronal cell loss and astrocyte hypotrophy in neuron-astrocyte co-cultures treated with 25 μM glutamate, which is significantly reduced in similarly treated tri-cultures (Fig. 4a and Supplementary Figure 4). As glutamate concentrations increased, astrocytes in the co-culture became progressively more reactive, evidenced by increasing hypertrophy and loss of processes. This resulted in a significant increase in astrocyte surface area following treatment with 10 μM and 25 μM glutamate, which is not observed in the tri-culture (Fig. 4b and Supplementary Data Table 1, *p* = 0.0022 and *p* = 0.0010, respectively). In order to quantify neuronal cell viability, we compared the percent area of the field-of-view stained for β-III tubulin with a circularity less than 0.2. The circularity cutoff was used to eliminate cell debris from apoptotic/necrotic neurons, which still stained for β-III tubulin, but lacked long cellular processes, and therefore had high circularity values. Across all concentrations of glutamate that were tested (5, 10, and 25 μM), there was significantly more neuronal cell loss in the co-culture then in the tri-culture (Fig. 4c and Supplementary Data Table 2, *p* = 0.017, *p* = 0.0010, and *p* = 0.0017, respectively). Furthermore, we observed that the neuron percent area significantly decreased in the co-cultures upon exposure to increasing concentrations of glutamate. In contrast, significant decreases in the neuron percent area were observed in tri-culture only at the highest concentration of glutamate. Surprisingly, unlike exposure to LPS or mechanical trauma, the exposure of the tri-cultures to glutamate did not appear to change the morphology of the microglia (Fig. 4d). Specifically, the average microglia area did not change following treatment with different concentrations of glutamate (*p* = 0.81).

We also confirmed that the tri-culture was electrophysiologically active via calcium imaging. For both the co- and tri-cultures, we observed spontaneous calcium influxes at DIV 7. Additionally, we compared the response of both culture types to varying concentrations of glutamate (Fig. 4e and Supplementary Data Table 3). Analysis of the main effects did not identify a significant difference in the response of the tri-culture and co-cultures to different concentrations of glutamate (*p* = 0.78). Additionally, for both culture types, we observed a significant change in fluorescence intensity across a wide range of glutamate concentrations (*p* = 1.73 × 10^–15^) indicating that the neuroprotective effect was a result of the presence of microglia in the tri-culture and was not due to a depressed neuronal response to glutamate.

### Tri-culture cytokine profile

**Fig. 5:**
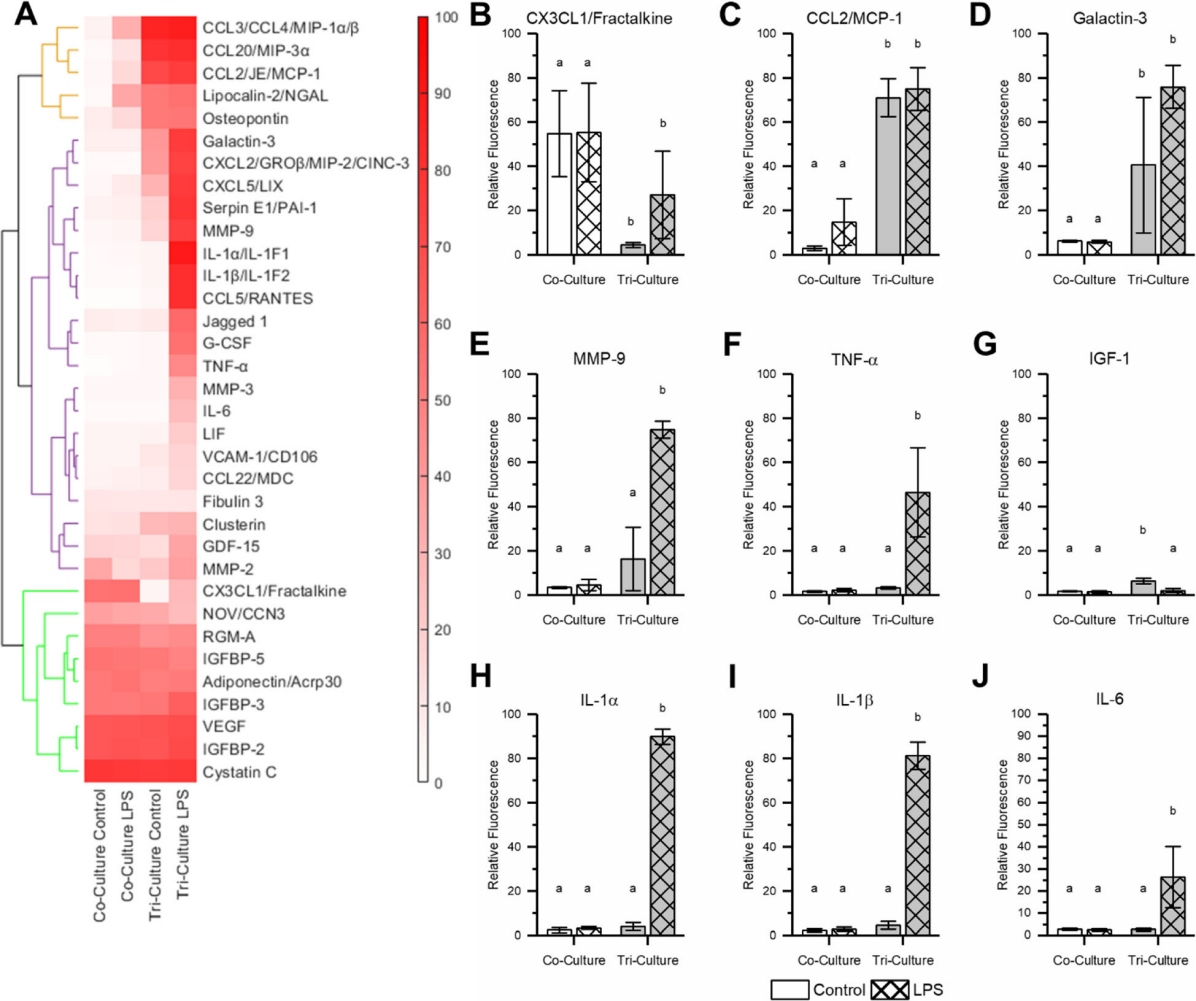
Tri-culture proteomic profile. Comparing the proteomic profile of the conditioned media from co- and tri-cultures after 48 h incubation with 5 μM LPS. **a** Heat map showing the relative cytokine concentrations scaled from 1 to 100. Proteins with a relative concentration of less than 10% of the maximum for all treatments were not included in the heat map but can be found in (Supplementary Figure 5). Hierarchical cluster analysis revealed three major groups of cytokines consisting of cytokines present in the conditioned media from all culture types (green), cytokines with increased expression in both control and LPS challenged tri-culture (orange), and cytokines with increased expression only in the LPS exposed tri-cultures (purple). **b**–**j** The relative concentrations of specific cytokines of interest. All graphs display mean ± SD (*n* = 3). The letters above the bars indicate statistically significant differences (*p* < 0.05, 2-way ANOVA with the Tukey test used to analyze the simple main effects if necessary). A full breakdown of the statistical analysis can be found in supplementary data table 4

We compared the cytokine secretion profiles of the control and LPS-exposed tri- and co-cultures using the proteome profiler rat XL cytokine array (Bio-Techne). Of the 79 cytokines detected by the array, 34 were detected at a relative concentration greater than 10% of the maximum in at least one of the samples. A hierarchical cluster analysis was performed on the results from these 34 cytokines (Fig. 5a and Supplementary Data Table 4), which revealed three distinct expression profiles. In general, these profiles consist of cytokines secreted by both the co- and tri-cultures (green) and those only secreted by the tri-culture. This second group of cytokines can be further subdivided into cytokines that are secreted in relatively equal concentrations by the control and LPS-exposed tri-cultures (orange) and cytokines having increased expression in the LPS-exposed tri-cultures (purple). As expected, the co-cultures did not respond to LPS, leading to a nearly indistinguishable cytokine profile as compared to the control co-culture exposed only to vehicle.

All cytokines secreted by both the tri- and co-cultures have been shown to be expressed by either neurons or astrocytes in non-inflammatory conditions [45–49], with the exception of adiponectin, which is found in the CSF of healthy individuals but has not been shown to be expressed by neurons or astrocytes [50]. Additionally, with the exception of CX3CL1, these cytokines appear to be constitutively expressed by astrocytes and microglia as neither the media type nor the addition of LPS had an impact on their concentration in the conditioned media. Interestingly, we see a significant decrease in CX3CL1 concentration in the tri-culture as compared to the co-culture (Fig. 5b, *p* = 0.0051). Additionally, we observe the presence of IGF-1 in the control tri-culture conditioned media that is not present in either the co-culture or the LPS-exposed tri-culture (Fig. 5g, *p* = 0.0010 and *p* = 0.0010, respectively).

Of the cytokines present in the tri-culture conditioned media that do not increase in expression in response to LPS, all have been shown to be secreted by microglia and are also linked to neuroinflammatory states [51–55]. In the case of CCL2, CCL3, and CCL20, the concentration of these cytokines in the conditioned media are at the upper limit of the array under control conditions; thus, an increase in concentration in response to LPS may not have been detected. However, there are a significant number of pro-inflammatory cytokines that are present in the conditioned media only after treatment with LPS, including many of the hallmark pro-inflammatory cytokines secreted by activated microglia. In particular, levels of TNF-α, IL-1α, IL-1β, and IL-6 are significantly increased in the conditioned media of tri-cultures challenged with LPS (Fig. 5f–j, *p* = 0.0033, *p* = 0.0010, *p* = 0.0010, *p* = 0.013, respectively). These four cytokines are consistently secreted by microglia in response to LPS in a variety of experimental conditions and are often used as biomarkers to indicate neuroinflammatory or neurodegenerative disorders [56].

## Discussion

Here, we describe an in vitro model consisting of a tri-culture of neurons, astrocytes, and microglia that more faithfully mimics neuroinflammatory responses than standard mono- and co-cultures. This tri-culture is established by culturing primary cortical cells from neonatal rats in a tri-culture medium supplemented with 100 ng/mL IL-34, 2 ng/mL TGF-β, and 1.5 μg/mL cholesterol. These three factors were chosen as they have been identified as key factors in supporting serum-free cultures of isolated microglia [19]. Previous studies have indicated that the activation of the colony-stimulating factor 1 receptor (CSF1R) via colony-stimulating factor 1 (CSF1) or IL-34 is required for microglia viability both in vitro and in vivo [57–59], and therefore, its requirement in the tri-culture media was not unexpected. While the addition of TGF-β or cholesterol did not seem to be a requirement for microglia viability in the tri-culture, both factors proved critical in maintaining physiologically active microglia. It has been shown that TGF-β can induce a quiescent microglia phenotype in vitro [60], and it may even be required for cultured microglia to maintain their gene expression profile [61]. Excess cholesterol in the culture media may also be beneficial for maintaining a functional microglia gene expression profile as it reduces the expression of apolipoprotein E (ApoE) [62], which is critical for lipid transport, but it is also inversely correlated with the expression of microglia signature genes [61].

We hypothesized that these three factors could be included in a culture medium designed to support neurons and astrocytes without negatively impacting the overall health of the culture, as each factor has been associated with improved neuronal health and survival. TGF-β enhances the effect neurotrophic factors and has been shown to improve the survival of cultured neurons maintained in low concentrations of neurotrophins [63, 64], while exogenous cholesterol is thought to play a significant role in neural cholesterol homeostasis [65]. Activation of CSF1R on neurons via CSF1 or IL-34 has been shown to increase neuron survival following kainic acid-induced excitotoxicity [44], while activation via CSF1 reduces apoptosis in cultured neurons in a dose-dependent manner [66]. We decided to include IL-34 instead of CSF1 in the tri-culture medium, as IL-34 is more widely expressed in the cortex of postnatal mice [67, 68]. We observed that the tri-culture contains all three cell types and that the continuous presence of microglia did not impact the number of neurons or neurite outgrowth. However, we did observe a slight decrease (*p* = 0.062) in the number of astrocytes in the tri-culture as compared to the co-culture, which might be due to the presence of TGF-β in the tri-culture media, which has been shown to reduce astrocyte proliferation [69]. Additionally, we observed a significant increase in the percent area coverage of the astrocytes at DIV 14. However, this increase is due in part to the large number of fine astrocyte processes seen in the DIV 14 tri-culture, which is overvalued by the auto-thresholding process used to calculate these values. We also observed a small portion of cells in both the tri- and co-cultures (~ 2%) that were not immunoreactive for antibodies that selectively label neurons, astrocytes, or microglia (Supplementary Figure 6a). These unidentified cells may be oligodendrocyte precursor cells based on immunoreactivity for NG2 (Supplementary Figure 6b) [70]. It is likely that the lack of a mitotic inhibitor may have allowed a small number of these cells to survive, as they are not observed in neuron-astrocyte co-cultures treated with mitotic inhibitors [21]. Furthermore, we do not expect that the presence of NG2-glia would impact the observed differences in the inflammatory response between the co- and tri-cultures as both cultures have a similar population of NG2-immunopositive cells. However, as there is evidence that NG2-glia can play a role in neuroinflammation [70–72], in future studies, it may be beneficial to attempt to increase the number of NG2-glia to physiologically relevant levels to attain an even more complete cell culture model of neuroinflammation.

The microglia in the tri-culture display an amoeboid morphology and not the classical ramified morphology associated with phenotypically “resting” microglia in the adult CNS. The cytokine profile of the tri-culture does contain molecules associated with neuroinflammatory states [51–55], which suggests that the tri-culture is in a slightly inflamed state, possibly due to trauma associated with dissection to isolated cells from the intact brain. However, we believe that the amoeboid morphology of the microglia is primarily due to the fact that the primary microglia present in the culture are derived from neonatal rats and therefore are still displaying their neonatal morphology and phenotype [73–75]. This hypothesis is supported by the presence of galactin-3 in the tri-culture (Fig. 5d). Galactin-3 has been shown to induce an amoeboid morphology in microglia, and the transcription factor for galactin-3 is highly expressed in healthy neonate amoeboid microglia, but not in adult microglia showing ramified morphologies [76]. Furthermore, IGF-1, which is produced by microglia in early postnatal mice, was also present in the tri-culture [77].

Crosstalk between different cell types in the CNS is integral in maintaining homeostasis, and we observe evidence of that crosstalk in the tri-culture. The tri-culture showed low concentrations of CX3CL1 in the conditioned media, which was elevated in both the co-culture conditions and the LPS exposed tri-culture. CX3CL1 is expressed primarily by neurons in the CNS and is found in both a membrane-bound and soluble form. Membrane-bound CX3CL1 is important for microglia regulation and thought to hold the microglia in resting state, while soluble CX3CL1 acts as a powerful chemoattractant for microglia and is also secreted by neurons and glial cells during neuroinflammatory conditions [11, 78, 79]. As microglia are an integral part of CNS homeostasis, the high concentration of CX3CL1 in the co-culture might be a compensatory response to the lack of microglia as co-cultures attempt to recruit them. The lack of CX3CL1 in the conditioned media from the control tri-cultures suggests that the majority of CX3CL1 in tri-cultures is membrane-bound and is interacting with the microglia in the tri-culture to hold them in a non-activated state. In response, the microglia secrete the neurotrophic factor, IGF-1 [77, 80], which was only found in the control tri-culture conditioned media. IGF-1 has been indicated as a trophic factor produced by microglia in postnatal (P3-7) mice that significantly increases the survival of layer V cortical neurons [77]. This crosstalk between neurons and microglia may have led to a healthier neural population, as we observe a significant decrease in caspase 3/7 activity in the tri-culture as compared to the co-culture (Supplementary Figure 3, *p* = 0.0031). However, we were unable to determine the extent to which the presence of microglia versus the additional factors present in the tri-culture medium improved neural health.

We characterized the tri-culture under a number of neuroinflammatory scenarios, including exposure to LPS, mechanical injury, and excitotoxic challenge. We compared the response of the tri-cultures to that of the neuron-astrocyte co-cultures and found that the presence of microglia changes the response to each of these neuroinflammatory stimuli. Moreover, the response of the tri-culture to these neuroinflammatory challenges are more in line with what is reported in vivo and in slice culture models.

In response to LPS, the tri-culture displays many classic hallmarks of neuroinflammation including an increase in caspase 3/7 activity, astrocyte hypertrophy, and the secretion of a number of pro-inflammatory cytokines. However, while we do see evidence of neurodegeneration and neurite loss in the LPS-exposed tri-cultures (Fig. 2a and Supplementary Figure 7A), the extent of the damage is significantly less severe than what is observed in vivo. This may be because microglia require co-activation with both LPS and INF-γ (secreted by circulating leukocytes) to produce the severe neurotoxic effects seen in vivo [81]. In the absence of leukocytes, no INF-γ is produced by the tri-culture in response to LPS (Supplementary Figure 5) leading to a less severe response. We also observe an increase in microglia area in response to LPS, contradictory to the decrease in microglia area typically observed in vivo as the microglia transition from a highly ramified to amoeboid morphology [82]. However, the morphology of the microglia following exposure to LPS is consistent with previous in vitro experiments, with the microglia showing a distinct polarity and the formation of large lamellipodia [30, 83, 84], which accounts for the increase in area. We believe that this difference may not be due to a difference in microglia function but a result of the change from a 3D to 2D environment. In both instances, microglia become activated and prepare to migrate towards the site of injury. In the 3D environment, this involves the transformation from a ramified to amoeboid morphology to allow for the free movement through the parenchyma, while in the 2D environment, this involves the extension of lamellipodia.

The neuroinflammatory response to the mechanical trauma caused by the implantation of therapeutic electrodes is a highly complex process, with both microglia and astrocytes responding to alarmins released by damaged cells to initiate an inflammatory cascade. Activated microglia display a wide range of phenotypes with overlapping gene expression profiles, and microglia polarization is no longer classified along a binary “M1 (neurotoxic)/M2 (neuroprotective)” scale based on the presence of specific markers [8, 85]. However, following the implantation of therapeutic electrodes, it is generally hypothesized that microglia play a pro-inflammatory and neurotoxic role through the secretion of reactive oxygen species and pro-inflammatory cytokines, such as TNF-α, IL-1β, and IL-6, along with the recruitment of circulating immune cells [6, 86]. The increased caspase 3/7 activity in the tri-culture culture 48 h following the scratch injury is in line with the hypothesized neurotoxic role of microglia during these types of injuries. Additionally, the tri-culture is capable of modeling the early stages of glial scarring, which is a major factor in the functional recovery following mechanical trauma [87, 88] and a primary reason for the loss of functionality of therapeutic neural implants [6, 89]. We observe a significantly higher coverage of astrocyte processes in the scratched area in the tri-culture as compared to the co-culture. In the tri-culture, we also find microglia in the scratched area, and these microglia appear to have a larger surface area, most likely due to the extension of lamellipodia as the microglia migrate into the scratched area. Microglia are thought to migrate into the injury site and secrete factors that lead to increased astrocyte migration towards the injury [88]. One such factor is matrix metalloprotease 9 (MMP-9), which is secreted by microglia in response to neuroinflammatory factors [90] and has been implicated in the migration of astrocytes and the initial formation of the glial scar [91]. We observed that MMP-9 is secreted in tri-cultures, but not in co-cultures in response to LPS (Fig. 5a).

The tri-culture model is especially useful in modeling the glial response to glutamate-induced excitotoxic events. Both astrocytes and microglia are thought to play a neuroprotective role during excitotoxic events through mechanisms that are difficult to capture in other models. Astrocytes are responsible for maintaining glutamate homeostasis in the CNS through the uptake of extracellular glutamate by membrane transporters, and the failure of these transporters is linked to excitotoxic pathologies [92, 93]. Furthermore, blocking these glutamate transporters with DL-threo-β-benzyloxyaspartic acid (TBOA) leads to excitotoxicity-induced neural death in otherwise healthy hippocampal slices [94] and increased neural death in neuron-astrocyte co-cultures exposed to glutamate [95]. While the exact mechanisms responsible for the neuroprotective effect of microglia in response to excitotoxicity are not fully understood, it has been shown that the microglia migrate towards hyperactive neurons in response to ATP and glutamate released by these neurons [96–98]. These microglia have been shown to release small quantities of TNF-α that protect neurons against NMDA-induced excitotoxicity [99], and to directly contact damaged axons to repolarize and rescue hyperactive neurons [98]. We observed a significant decrease in the number of neurons lost in the tri-culture as compared to the co-culture following exposure to glutamate (Fig. 4c and Supplementary Figure 7B), indicating that the tri-culture recapitulates the complex interactions between the microglia and neurons during excitotoxicity. Additionally, we observed that the average microglia area did not change in response to exposure to different concentrations of glutamate, which is in line with previous research suggesting morphologically resting/ramified microglia can serve a neuroprotective role during excitotoxic events [97].

We believe the tri-culture model has multiple advantages over other methods used to study neuroinflammation in vitro*.* The most obvious benefit is the presence of neurons, astrocytes, and microglia in the same cell culture, which will allow researchers to study the complex interplay between these cells that leads to different responses to inflammatory stimuli. Additionally, the use of a single culture (as opposed to using conditioned media to mimic the influence of cell types absent in the culture) allows for the observation of cell-cell interactions and other mechanisms with spatiotemporal nuances that may be lost when using models involving multiple different types of cultures. Another major benefit of the tri-culture model is its relative simplicity. The only modification needed to establish and maintain the tri-culture model is the use of a specialized tri-culture medium. Compared to other models that involve multiple cultures or the addition of cells at specific time points during culture, the simplicity of this tri-culture model lends itself to high-throughput experiments, and, therefore, it may be an effective tool in the early screening of potential therapeutic molecules. Additionally, this tri-culture model is amenable to experiments involving more complex culture setups, such as microfluidic and organ-on-a-chip devices [100]. Ultimately, we believe that the neuron, astrocyte, and microglia tri-culture described in this paper can be a useful tool to study neuroinflammation in vitro with improved accuracy in predicting in vivo neuroinflammatory phenomena.

## Conclusion

We have described a primary neural cell culture model consisting of a tri-culture of neurons, astrocytes, and microglia. The response of this tri-culture to LPS exposure, mechanical trauma, and glutamate-induced excitotoxicity more faithfully mimicked the in vivo response than an established neuron-astrocyte co-culture model. We believe that this tri-culture model can be a useful tool to study neuroinflammation in vitro.

## Supplementary information

**Additional file 1: Supplementary Data Table 1.** Analysis of the simple main effects (Tukey test) from Figure 4B. The p-values from each pairwise result are shown on the table, with p-values less than 0.05 highlighted in green. **Supplementary Data Table 2.** Analysis of the simple main effects (Tukey test) from Figure 4C. The p-values from each pairwise result are shown on the table, with p-values less than 0.05 highlighted in green. **Supplementary Data Table 3.** Analysis of the simple main effects (Tukey test) from Figure 4E. The p-values from each pairwise result are shown on the table, with p-values less than 0.05 highlighted in green. **Supplementary Data Table 4.** Statistical analysis of Figure 5B-J. The p-values from the 2-way ANOVA and simple main effects analysis (Tukey Test) are shown. p-values < 0.05 are highlighted in green. **Supplementary Figure 1.** Immunostained images of the co- and tri-cultures at DIV 14 showing the co-localization of f-actin (cyan) and β-III tubulin (red) indicative of dendritic spines. Scale bar = 20 μm. **Supplementary Figure 2.** Tri-culture media supplement requirements for microglia survival at DIV 7. The results indicate that IL-34 is required for microglial survival in the tri-culture. The figure shows the mean ± SD of the technical replicates (n = 4) of a single biological replicate. **Supplementary Figure 3.** The tri-culture shows reduced caspase 3/7 activity at DIV 9 (n = 6). The letters above the bars indicate statistically distinct groups (p < 0.05), while the points indicate the values of the technical replicates. **Supplementary Figure 4.** Representative images of the co- and tri-cultures 48 h following a 1 h treatment with different concentrations of glutamate or vehicle control. The cultures were immunostained for the three cell types of interest: neurons – anti-βIII-tubulin (red), astrocytes – anti-GFAP (green), microglia – anti-Iba1 (orange) and the general nuclear stain DAPI (blue). Scale bar = 100 μM. **Supplementary Figure 5** Complete proteomic profile from Figure 5A. **Supplementary Figure 6.** There are approximately 2% of the total cell population that was not clearly identifiable as neurons, astrocytes or microglia. (**A**) Mean ± SD of cells from each culture type not reactive for antibodies selective for neurons, astrocytes or microglia (n = 3). (**B**) Representative images from DIV 7 co- and tri-cultures immunostained for NG2 (red), a biomarker of oligodendrocyte precursor cells (OPCs), and reacted with DAPI (blue), scale bar = 100 μm. **Supplementary Figure 7.** Change in number of cells following incubation with LPS or 25 μM glutamate. (**A**) Percent change in cell number following incubation with LPS. (**B**) Percent change in cell number following incubation with 25 μM glutamate. All graphs display mean ± SD (*n* = 3).

## Data Availability

The datasets during and/or analyzed during the current study are available from the corresponding author on reasonable request.

## Abbreviations

LPS: Lipopolysaccharide
IGF-1: Insulin-like growth factor 1
CX3CL1: Chemokine (C-X3-C motif) ligand 1 (fractalkine)
TNF-α: Tumor necrosis factor
IL: Interleukin
CNS: Central nervous system
DIV: Days in vitro
DPBS: Dulbecco’s phosphate-buffered saline solution
DPBS+: Dulbecco’s phosphate-buffered saline solution with calcium and magnesium
PBS: Phosphate-buffered saline solution
PFA: Paraformaldehyde
GFAP: Glial fibrillary acidic protein
Iba-1: Ionized calcium-binding adaptor molecule 1
DAPI: 4′,6-Diamidino-2-phenylindole
ANOVA: Analysis of variance
TLR4: Toll-like receptor 4
TGF-β: Transforming growth factor beta
AD: Alzheimer’s disease
PD: Parkinson’s disease
CCL: Chemokine (C-C motif) ligand
CSF1: Colony-stimulating factor 1
CSF1R: Colony-stimulating factor 1 receptor
INF-γ: Interferon gamma
TBOA: DL-threo-β-benzyloxyaspartic acid
ATP: Adenosine triphosphate
NMDA: N-methyl-d-aspartate
