# Correction to: A primary neural cell culture model to study neuron, astrocyte, and microglia interactions in neuroinflammation

**DOI:** 10.1186/s12974-022-02391-4

**Published:** 2022-02-12

**Authors:** Noah Goshi, Rhianna K. Morgan, Pamela J. Lein, Erkin Seker

**Affiliations:** 1 Department of Biomedical Engineering, University of California-Davis, Davis, CA 95616 USA; 2 Department of Molecular Biosciences, University of California-Davis, Davis, CA 95616 USA; 3 Department of Electrical and Computer Engineering, University of California-Davis, 3177 Kemper Hall, Davis, CA 95616 USA

## Correction to: Journal of Neuroinflammation (2020) 17:155 https://doi.org/10.1186/s12974-020-01819-z

Following publication of the original article [1], the authors identified a typo in one value. The 5 μg/mL as the LPS concentration were wrongly given as 5 μM in several instances in the article. This typo does not affect any results or conclusions.

It should read as: 5 μg/mL.
