# Supplementary material for: A primary neural cell culture model to study neuron, astrocyte, and microglia interactions in neuroinflammation
Source: J Neuroinflammation. 2020 May 11;17:155. doi: 10.1186/s12974-020-01819-z (PMC7216677; doi:10.1186/s12974-020-01819-z)
Supplement: Supplementary file 1 — Additional file 1: Supplementary Data Table 1. Analysis of the simple main effects (Tukey test) from Figure 4B. The p-values from each pairwise result are shown on the table, with p-values less than 0.05 highlighted in green. Supplementary Data Table 2. Analysis of the simple main effects (Tukey test) from Figure 4C. The p-values from each pairwise result are shown on the table, with p-values less than 0.05 highlighted in green. Supplementary Data Table 3. Analysis of the simple main effects (Tukey test) from Figure 4E. The p-values from each pairwise result are shown on the table, with p-values less than 0.05 highlighted in green. Supplementary Data Table 4. Statistical analysis of Figure 5B-J. The p-values from the 2-way ANOVA and simple main effects analysis (Tukey Test) are shown. p-values < 0.05 are highlighted in green. Supplementary Figure 1. Immunostained images of the co- and tri-cultures at DIV 14 showing the co-localization of f-actin (cyan) and β-III tubulin (red) indicative of dendritic spines. Scale bar = 20 μm. Supplementary Figure 2. Tri-culture media supplement requirements for microglia survival at DIV 7. The results indicate that IL-34 is required for microglial survival in the tri-culture. The figure shows the mean ± SD of the technical replicates (n = 4) of a single biological replicate. Supplementary Figure 3. The tri-culture shows reduced caspase 3/7 activity at DIV 9 (n = 6). The letters above the bars indicate statistically distinct groups (p < 0.05), while the points indicate the values of the technical replicates. Supplementary Figure 4. Representative images of the co- and tri-cultures 48 h following a 1 h treatment with different concentrations of glutamate or vehicle control. The cultures were immunostained for the three cell types of interest: neurons – anti-βIII-tubulin (red), astrocytes – anti-GFAP (green), microglia – anti-Iba1 (orange) and the general nuclear stain DAPI (blue). Scale bar = 100 μM. Supplementary Figure 5 Complete pr [file 12974_2020_1819_MOESM1_ESM.docx]

Supporting Information for:

**A Primary Neural Cell Culture Model to Study Neuron, Astrocyte and Microglia Interactions in Neuroinflammation**

Noah Goshi^1^, Rhianna K. Morgan^2^, Pamela J. Lein^2^, Erkin Seker^3^

Departments of ^1^Biomedical Engineering, ^2^Molecular Biosciences, and ^3^Electrical and Computer Engineering, University of California - Davis, Davis, CA 95616


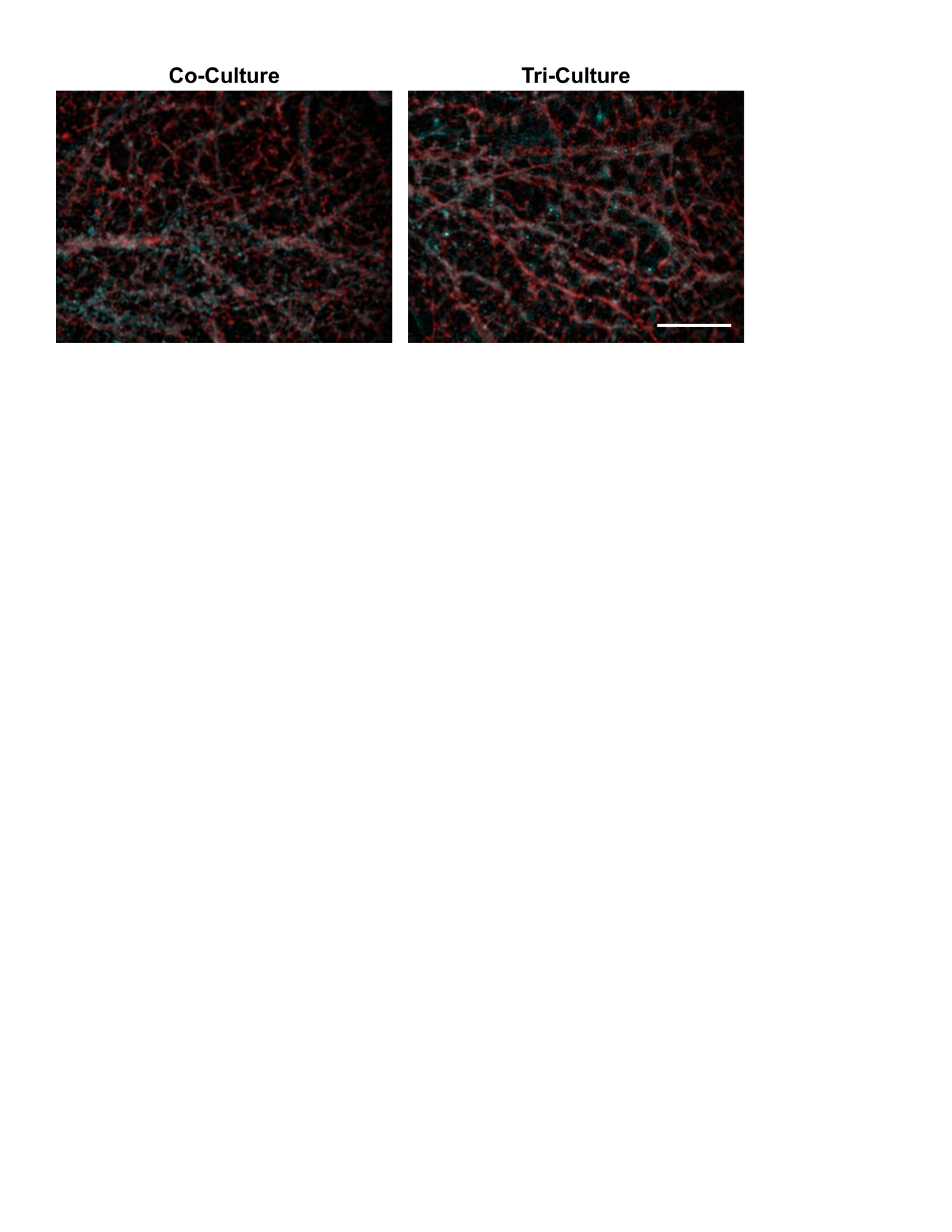


**Supplementary Figure 1:** Immunostained images of the co- and tri-cultures at DIV 14 showing the co-localization of f-actin (cyan) and β-III tubulin (red) indicative of dendritic spines. Scale bar = 20 µm.





**Supplementary Figure 2:** Tri-culture media supplement requirements for microglia survival at DIV 7. The results indicate that IL-34 is required for microglial survival in the tri-culture. The figure shows the mean ± SD of the technical replicates (n = 4) of a single biological replicate.





**Supplementary Figure 3:** The tri-culture shows reduced caspase 3/7 activity at DIV 9 (n = 6). The letters above the bars indicate statistically-distinct groups (p < 0.05), while the points indicate the values of the technical replicates.

**Supplementary Data Table 1:** Analysis of the simple main effects (Tukey test) from Figure 4B. The p-values from each pairwise result are shown on the table, with p-values less than 0.05 highlighted in green.

**Supplementary Data Table 2:** Analysis of the simple main effects (Tukey test) from Figure 4C. The p-values from each pairwise result are shown on the table, with p-values less than 0.05 highlighted in green.


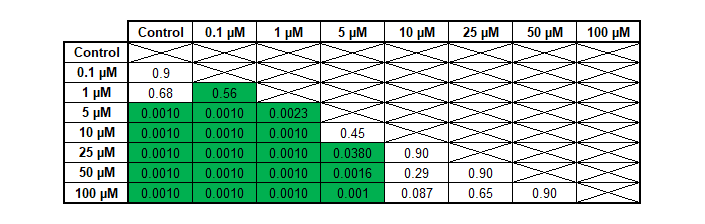


**Supplementary Data Table 3:** Analysis of the simple main effects (Tukey test) from Figure 4E. The p-values from each pairwise result are shown on the table, with p-values less than 0.05 highlighted in green.


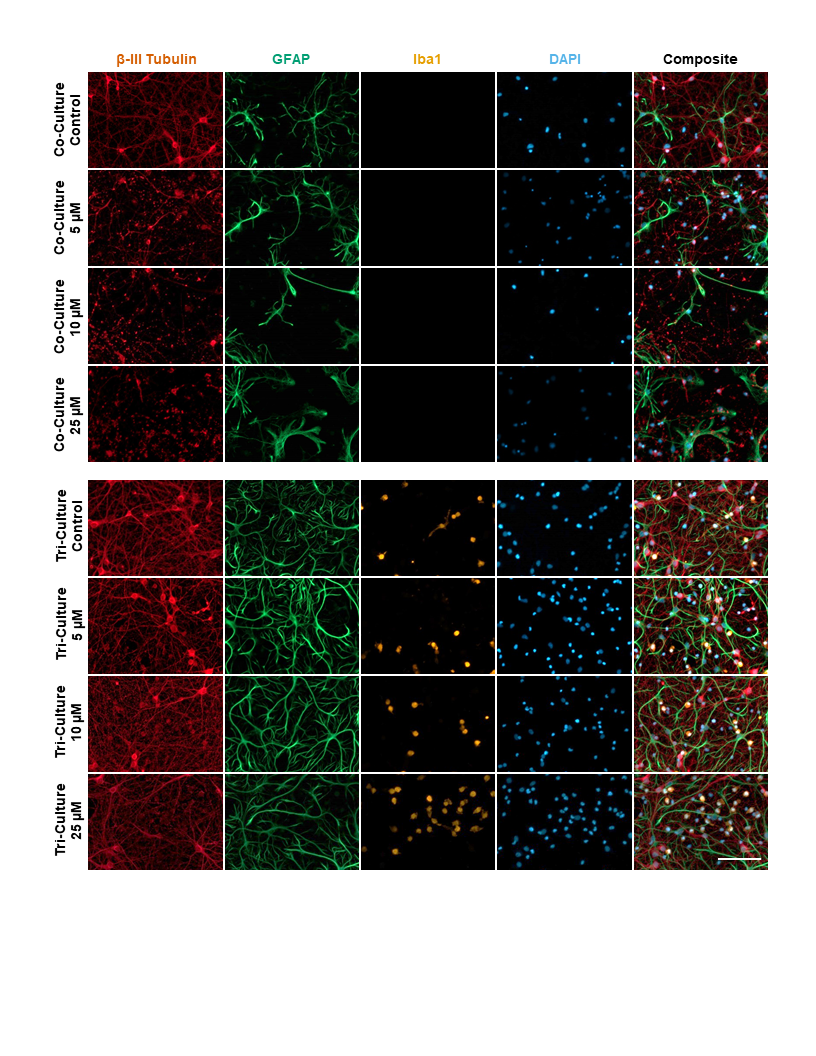


**Supplementary Figure 4:** Representative images of the co- and tri-cultures 48 h following a 1 h treatment with different concentrations of glutamate or vehicle control. The cultures were immunostained for the three cell types of interest: neurons – anti-βIII-tubulin (red), astrocytes – anti-GFAP (green), microglia – anti-Iba1 (orange) and the general nuclear stain DAPI (blue). Scale bar = 100 µM.


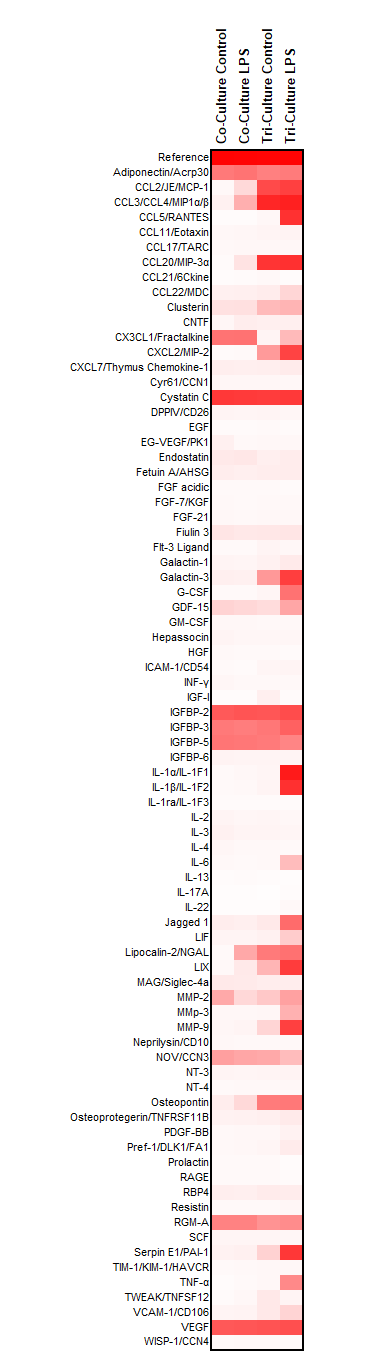


**Supplementary Figure 5:** Complete cytokine profile from Figure 5A.

**Supplementary Data Table 4:** Statistical analysis of Figure 5B-J. The p-values from the 2-way ANOVA and simple main effects analysis (Tukey Test) are shown. p-values < 0.05 are highlighted in green.

**
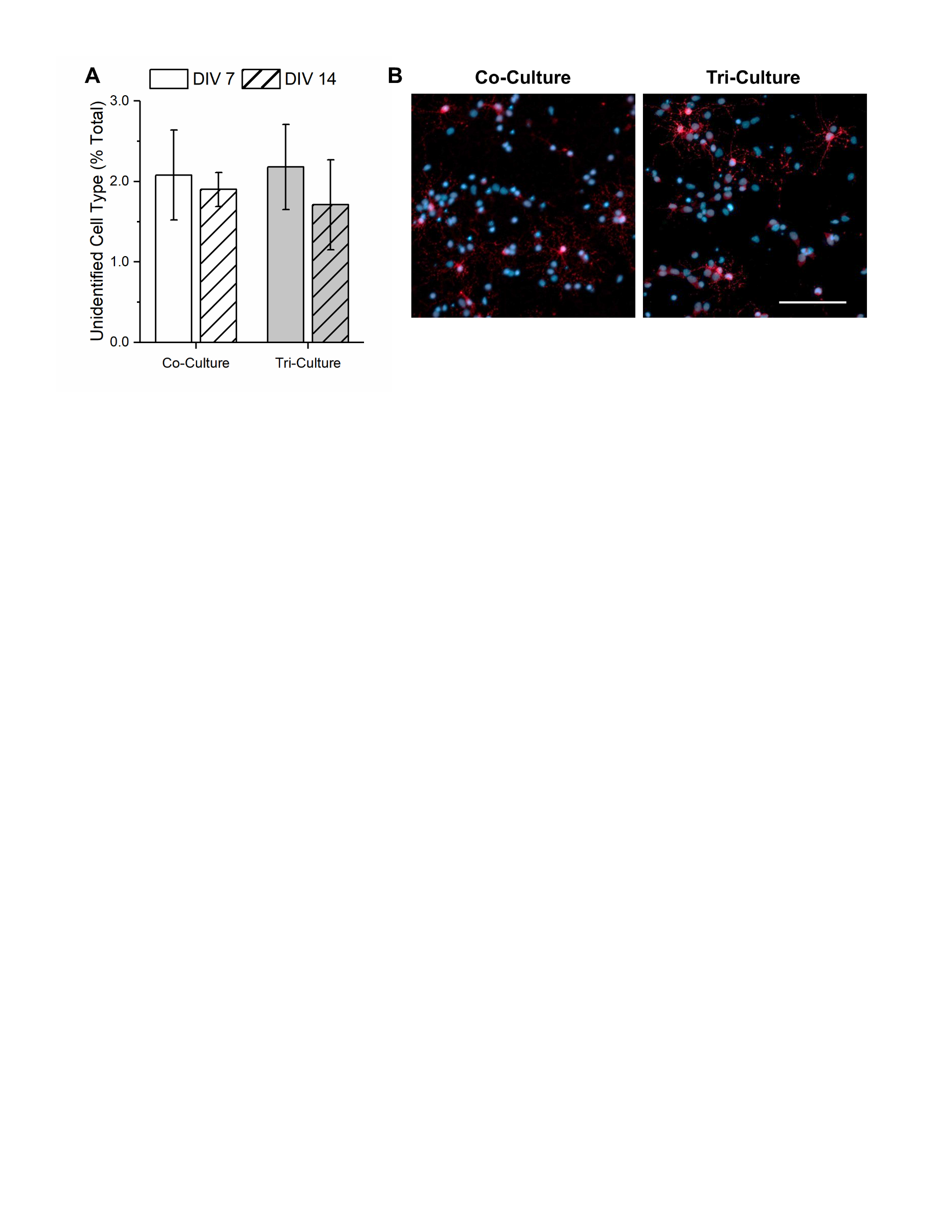
**

**Supplementary Figure 6:** There are approximately 2% of the total cell population that was not clearly identifiable as neurons, astrocytes or microglia. (**A**) Mean ± SD of cells from each culture type not reactive for antibodies selective for neurons, astrocytes or microglia (n = 3). (**B**) Representative images from DIV 7 co- and tri-cultures immunostained for NG2 (red), a biomarker of oligodendrocyte precursor cells (OPCs), and reacted with DAPI (blue), scale bar = 100 µm.


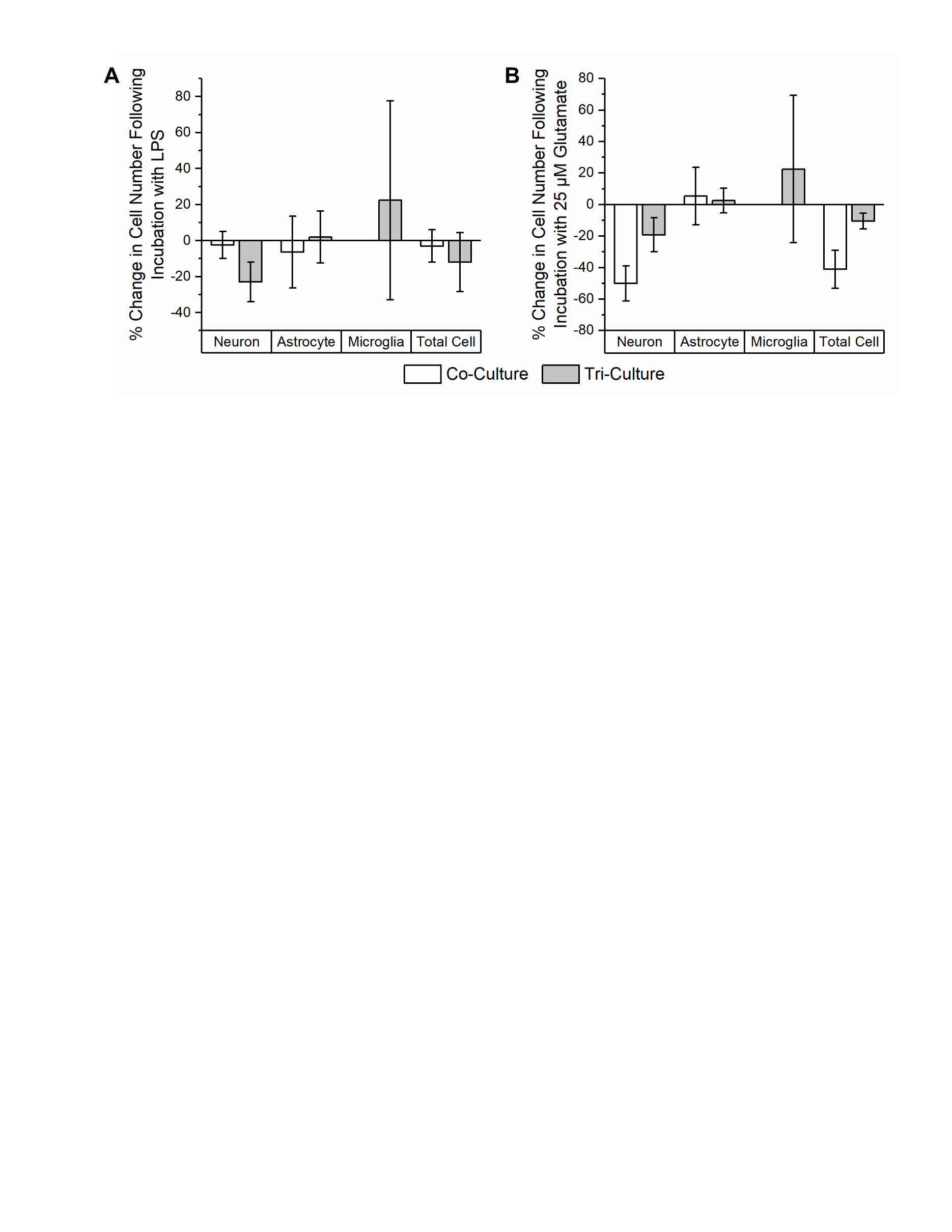


**Supplementary Figure 7:** Change in number of cells following incubation with LPS or 25 µM glutamate. (**A**) Percent change in cell number following incubation with LPS. (**B**) Percent change in cell number following incubation with 25 µM glutamate. All graphs display mean ± SD (n = 3).
